# Thermal and Catalytic Recycling of Plastics from Waste Electrical and Electronic Equipment—Challenges and Perspectives

**DOI:** 10.3390/polym16172538

**Published:** 2024-09-07

**Authors:** Dimitris S. Achilias, Maria-Anna Charitopoulou, Stefano Vecchio Ciprioti

**Affiliations:** 1 Laboratory of Polymer and Colours Chemistry and Technology, Department of Chemistry, Aristotle University of Thessaloniki, 54124 Thessaloniki, Greece; ccmariaa@chem.auth.gr; 2 Department of Basic and Applied Sciences for Engineering, Sapienza University of Rome, 00161 Rome, Italy; stefano.vecchio@uniroma1.it

**Keywords:** WEEE, thermal recycling, catalytic recycling, plastics, pyrolysis

## Abstract

The amount of end-of-life electrical and electronic devices has been widely increased, globally. This emphasizes how recycling waste electric and electronic equipment (WEEE) is essential in order to reduce the amount of WEEE that is disposed of directly in the environment. Plastics account for a big percentage in WEEE, almost 20%. As a result, the application of recycling methods on plastics gathered from WEEE is of great importance since, in this way, landfill disposal can be reduced. Nevertheless, despite the advantages, there are a lot of difficulties, such as the variety of different plastics present in the plastic mix and the existence of various additives in the plastic parts, for instance, brominated flame retardants that need special attention during their treatments, which restricts their wide application. Considering all these, this review aims to provide readers with all the current techniques and perspectives that are available for both the thermal and the catalytic recycling of plastics retrieved from WEEE. Apart from the up-to-date information on the recycling methods, in this review, emphasis is also given on the advantages each method offers and also on the difficulties and the limitations that may prevent them from being applied on a large scale. Current challenges are critically examined, including the use of mechanical or thermo-chemical recycling, the treatment of individual polymers or polymer blends and the separation of harmful additives before recycling or not. Finally, emerging technologies are briefly discussed.

## 1. Introduction

**Figure 1:**
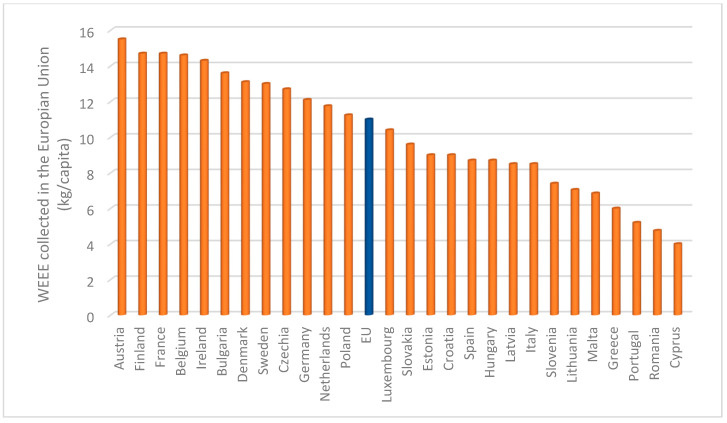
Waste electrical and electronic equipment collected in the European Union in 2021 [4].

Over the last few decades, better living conditions, along with the short lifespan of electrical and electronic equipment (EEE), have led to a rise in EEE production and consumption, resulting in huge amounts of post-consumer devices [1]. As the market continues to expand, innovation cycles become even shorter, and so the replacement of equipment is accelerated, making EEE one of the fastest growing sources of waste [2]. Indicatively, it should be highlighted that the annual generation of waste electrical and electronic equipment (WEEE) is increasing by 3–5% [3]. According to Eurostat, approximately 11.0 kg of waste electrical and electronic equipment were collected per inhabitant in the European Union during 2021. Figure 1 presents in detail the kg per inhabitant of WEEE collected for each European country [4].

**Figure 2:**
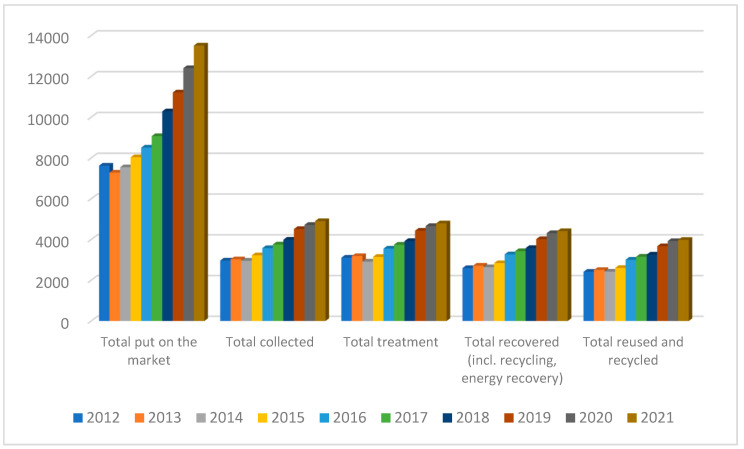
Amounts of EEE in thousand tones found on the European market along with the amounts processed from 2012 to 2021 [4].

As a consequence, the effective management of WEEE is of paramount importance and is regulated by the latest Directive 2012/19/EU of the European Parliament (WEEE Directive), which has been in effect until now. The first WEEE Directive (Directive 2002/96/EC) came into effect in February 2003 and was replaced by Directive 2012/19/EU, where increased collection targets were set [4]. However, as it can be seen in Figure 2, the amounts of WEEE that are reused and recycled are quite limited compared with the amounts found on the market. Therefore, on 6 October 2023, the European Commission recommended an improvement in the rate that used and waste mobile phones, tablets, and laptops are returned [5].

**Figure 3:**
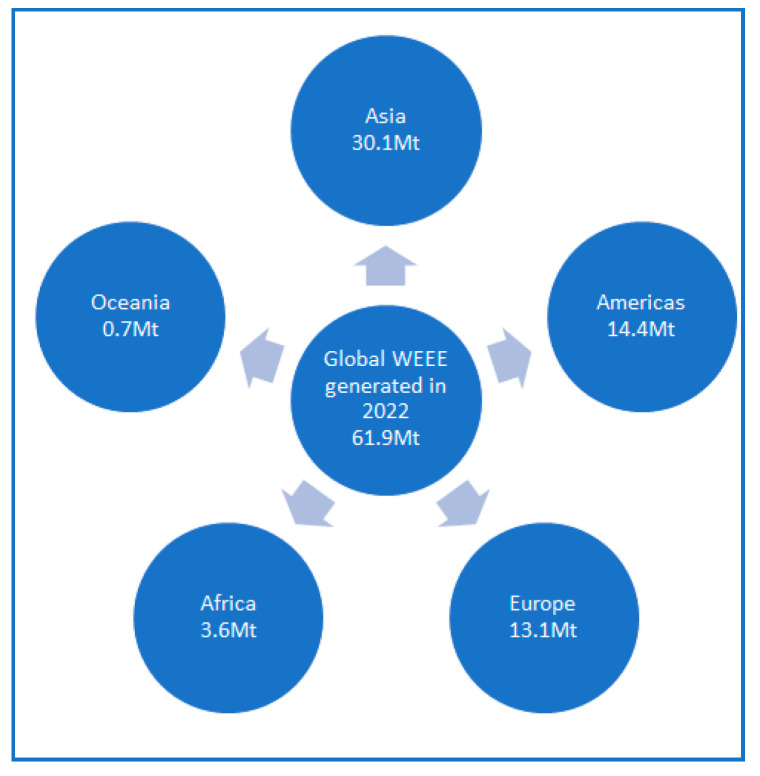
Quantities of WEEE generated during 2022 globally and per continent [6].

Apart from Europe, it is observed that globally, the amount of WEEE generated per year has greatly increased due to the aforementioned technological progress, the increasing consumption rates, the limited repair options available, as well as inadequate waste management [6,7]. Specifically, the worldwide amount of WEEE almost doubled from 2010 to 2022 since it grew from 34 billion kg in 2010 to ~62 billion kg in 2022. The WEEE generated varied among the different continents (Figure 3). For instance, during 2022, in Asia, ~30.1 billion kg of WEEE was generated (in comparison to ~13.3 billion kg generated during 2010); likewise, in the Americas, the amount was ~14.4 billion kg in 2022 (if compared with ~9.1 billion kg generated during 2010); in Europe, the total WEEE generated during 2022 was ~13.1 billion kg (instead of ~9.7 billion kg in 2010); and lower amounts were observed in Africa and Oceania [6].

Therefore, research on WEEE and its appropriate handling has increased around the world over the last few years, something that was proved by a bibliometric analysis carried out by Zhang et al., 2019 [8]. Studies focus on various recycling methods, such as primary recycling, energy recovery, mechanical recycling and chemical recycling [9]. Some studies investigate WEEE’s negative impacts both on humans and the environment [10,11], taking into account the fact that WEEE is a complex mixture of materials, some of which are hazardous and capable of causing serious environmental and health issues when discarded devices are not managed appropriately [12]. Specifically, WEEE comprises various and frequently useful materials, such as metals, glass, plastics, etc., that could be reused [13], especially modern electronic devices that consist of rare and expensive resources, such as critical raw materials, that may be recycled and re-used when WEEE is effectively managed [12].

Plastics only account for almost 30% of WEEE and contain various hazardous additives, such as heavy metals (for instance, lead, mercury, and hexavalent chromium) and others, including flame retardants [14], mainly brominated or chlorinated flame retardants [15]. Due to the non-negligible percentage of plastics found in WEEE, many studies investigate only the recycling of the plastic part of WEEE. One widely examined method is pyrolysis (belonging to chemical recycling methods), either thermal or catalytic, which aims to obtain secondary valuable materials or even monomers [14]. Since pyrolysis is considered as an advantageous technique for the recycling of plastics in WEEE, many studies have been carried out on real plastic waste, such as printed circuit boards (PCBs), modem Wi-Fi plastics, computers, televisions, remote controls, printers, calculators, tablets and mobile phones, etc. [16,17,18,19], in order to optimize the conditions and the products formed.

Nevertheless, fully comprehensive and up-to-date reviews are quite limited, and this review aims to fill this gap by studying in detail all the current methods that can be applied for the recycling of plastics originating in WEEE. For this reason, both thermal and catalytic techniques will be investigated; emphasis will be given on pyrolysis since it is considered a promising and fruitful method, during which monomers and other valuable materials may be received in order to be used again for the production of new polymeric materials. In this way, the principles of circular economy can be met. Finally, but importantly, not only the advantages but also all the possible difficulties and limitations associated with every technique presented will be fully analyzed.

To perform this review several papers were considered from international scientific bases, such as SCOPUS, using specific keywords, e.g., “thermal re-cycling”, “catalytic recycling”, “WEEE”, and “plastics” or “polymers”. From the large number of papers returned, to include/exclude papers, we did not follow any specific method or process but the more relevant and recent were selected to be commented on here.

## 2. Definition, Categories and Composition of WEEE

### 2.1. Definition of EEE and WEEE

According to the Directive (2012/19/EU), a definition for electrical and electronic equipment (EEE) could be the following: equipment that depends on electric currents or electromagnetic fields in order to work properly and perform the function for which it was designed. It should be highlighted here that EEE involves the entire equipment, including all components, sub-assemblies, accessories, and consumables (while of course excluding packaging, batteries, instructions, and manuals) [2]. As a result, a large range of devices are included in EEE, from mobile phones to photovoltaic panels [12], and can be divided into different categories based on their size or use.

Waste electrical and electronic equipment (WEEE) refers to all electrical or electronic equipment that is waste, including again all components, sub-assemblies and consumables that are part of the device during the time of discarding [2].

### 2.2. Categories of EEE

The main categories that EEE (and so WEEE) falls into, according to the Directive, are the following [2]:

- **Large household appliances** (including devices large in size, such as refrigerators, washing machines, electric heating appliances, air conditioner appliances, etc.);
- **Small household appliances** (including devices smaller in size, such as irons, toasters, coffee machines, electric knives, etc.);
- **IT and telecommunications equipment** (such as personal and laptop computers, with all accessories: CPU, mouse, screen and keyboard included, printers, copying equipment, pocket and desk calculators, telephones and cellular telephones, etc.);
- **Consumer equipment and photovoltaic panels** (such as radio sets, television sets, video cameras, photovoltaic panels, etc.);
- **Lighting equipment** (different types of lamps, such as straight fluorescent or compact fluorescent, and others);
- **Electrical and electronic tools** (such as drills, saws, sewing machines, etc., with the exception of large-scale stationary industrial tools);
- **Toys and leisure and sports equipment** (for instance, electric trains, video games, etc., and sports equipment with electric or electronic components);
- **Medical devices** (for instance, radiotherapy equipment, cardiology equipment, etc., with the exception of all implanted and infected products);
- **Monitoring and control instruments** (such as smoke detectors, heating regulators, thermostats and others);
- **Automatic dispensers** (for instance, automatic dispensers for hot drinks, automatic dispensers for money, and all appliances that deliver automatically all kinds of products).

### 2.3. Composition of WEEE

WEEE is highly non-homogenous, since it is a complex mixture of different materials and components, some of which may be hazardous or even toxic [9,20]. For instance, heavy metals (such as lead (Pb), mercury (Hg), cadmium (Cd), antimony (Sb), etc.) [21], brominated flame retardants (BFRs), refrigerants, etc., are substances that require safe handling so as to avoid environmental issues. Apart from toxic compounds, WEEE contains many valuable materials, such as metals, glass and plastics, which can be recovered from waste [9]. Among the metals found in WEEE, the most precious are silver (Ag) and gold (Au), followed by copper (Cu), zinc (Zn), and others [22].

Generally, WEEE’s composition varies, depending on the design and the use of each device; nevertheless, the materials found in WEEE can be divided into the following five categories [3]:

- Ferrous metals;
- Non-ferrous metals;
- Glass;
- Plastics;
- Other materials.

**Figure 4:**
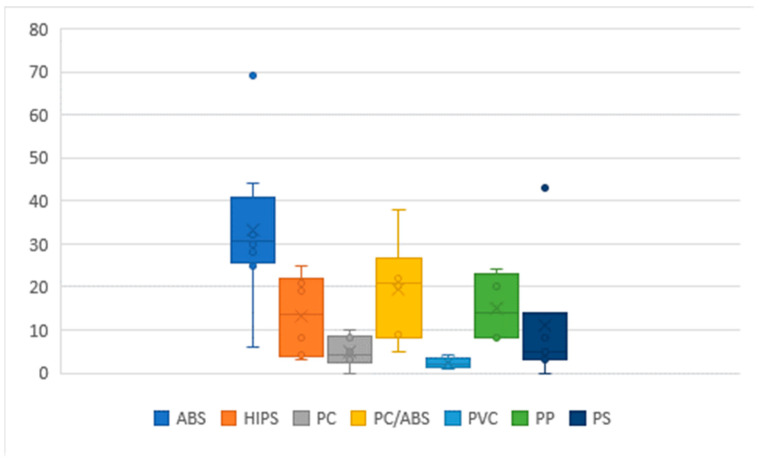
Range of plastic composition measured in WEEE.

In WEEE, ferrous metals account for approximately 50%, non-ferrous metals for ~5%, refractory oxides for 20–40 wt.% and plastics for 20–30 wt.% [23,24]. Plastics’ properties such as its light weight, durability, ease of processing and design versatility, along with the fact that they can be used as insulators, render them essential components in electrical and electronic devices [23,25,26]. Common polymers identified in WEEE are acrylonitrile-butadiene-styrene (ABS), high-impact polystyrene (HIPS), polycarbonate (PC), polyolefins [polyethylene (PE) and polypropylene (PP)], polystyrene (PS), polyamides (PAs), poly (vinyl chloride) (PVC) [25], etc., as well as blends of some of them (for instance, PC/ABS) [9]. Figure 4 presents a range of polymer compositions found in WEEE, estimated from Refs. [7,14,27,28,29]. As it is shown, in most applications—the most dominant polymers in WEEE are usually ABS, HIPS, PC, a blend of PC/ABS, PVC, PS, and PP [9]. In a recent study [7], the mean composition of polymers in different types of WEEE was reported as 25% ABS, 24% PP, 19% HIPS, 3% PC, 5% PC/ABS, 5% PUR, 2% PBT, and 1.5% PVC. Nevertheless, the type of the polymer/polymers (blend) used depends on the device and its use; for instance, samples of printers or computers usually consist of PS, while samples of cables or wires comprise PC [17,23]. In addition, additives to darken the color make the identification of polymers difficult [29].

#### Hazardous Substances in WEEE and Legislation

In spite of the fact that legislation has become stricter over the last few years (the RoHS Directive), hazardous compounds are still present in EEE and therefore in WEEE. The aim of the RoHS Directive is to protect human health and the environment from risks associated with the management of electronic and electrical waste by restricting the use of some specific hazardous substances in EEE, which can be substituted by safer alternatives. Such restricted substances contain heavy metals and flame retardants or plasticizers that are often added in plastics in EEE in order to improve their properties. Specifically, the RoHS Directive has restricted the use of lead (Pb), mercury (Hg), cadmium (Cd), hexavalent chromium (Cr VI), polybrominated biphenyls (PBBs), and polybrominated diphenyl ethers (PBDEs). PBB and PBDE are used as flame retardants in plastics that are found in a great variety of domestic objects, such as computers, TVs, etc., but are harmful to the environment and dangerous for the health of wildlife and probably humans [24,30]. The aforementioned brominated flame retardants (BFRs) are listed in the Stockholm Convention as persistent organic pollutants (POPs) due to their characteristics (long-range transportation, bio-accumulation, persistence in the environment, and toxicity); so, they must be separated from the rest of the plastic fractions [31,32].

Nevertheless, in spite of the RoHS restriction, hazardous substances are still present in WEEE. One main reason is the fact that the legislation was applied to all new EEE put on the market from 1 July 2006; as a result, all “older” WEEE consists of pre-RoHS waste and may comprise these restricted compounds. Furthermore, other hazardous substances that are not restricted under the current RoHS Directive are now used as alternatives, including Tetrabromobisphenol A (TBBPA), hexabromocyclododecane (HBCD or HBCDD) and others [24].

Several European projects have been funded by the European Union’s Horizon 2020 research and innovation program aiming at the removal of hazardous flame retardants from plastic waste in WEEE [33]. In the CREAToR project, the plastics targeted were ABS/PC, ABS, and HIPS and the elements were heavy metals, including Cd, Pb and Cr, BFRs and chlorinated flame retardants (CFRs). The objective of the PLAST2bCLEANED project was focused on HIPS and ABS plastics. BFRs and Sb_2_O_3_ were recovered using superheated solvents. In the NON-TOX project, an automated highspeed sensor (AHS) was used for sorting plastics (80% efficiency was achieved in classifying BFRs). Additives were extracted while the polymer’s properties were maintained. Additionally, halogenated polymers released the halogens contained using thermochemical conversion at specific temperatures.

Apart from BFRs, other halogenated flame retardants (for instance, chlorinated retardants) can be used as well or even non-halogenated retardants, including phosphorus-based flame retardants, nitrogen-based flame retardants and inorganic flame retardants [15]. However, BFRs are the most common flame retardants applied to EEE plastics. TBBPA and HBCD are now the most widely used BFRs. TBBPA only accounts for more than 50% of the total BFR volume [9,28] and is often used as additive BFRs in ABS and HIPS, phenolic resins, etc. [34]. HBCD, on the other hand, is mainly used in polystyrene foams (extruded and expanded), crystal and high-impact polystyrene, etc. [35]. However, both of them present toxic characteristics and can be classified as POPs [36]. Halogenated flame retardants are often combined with synergists, including antimony oxide, zinc borate, etc., in order to be more efficient in the vapor phase [37].

Not only flame retardants but other chemical additives can be found in plastics from WEEE, such as UV and thermal stabilizers (so as to prevent degradation from heat and light [23]), antistatic agents, colorants, pigments, plasticizers, fillers, etc., with the aim of fulfilling specific functional requirements [22,38]. As a result, a great variety of chemical substances are used in plastics from WEEE that may, however, be released during the life cycle or the recycling of the plastic devices, endangering human health and the environment [39] and, in the meantime, enhancing the difficulties in WEEE’s separation and recycling [14].

## 3. Recycling of Plastics from WEEE

According to the European Electronic Recyclers Association [7,40], the “ideal current management scheme” is a scenario where the collected WEEE are all treated by means of the best options currently available for material recovery, when this is possible, and incineration for energy recovery, without any material sent to landfilling.

This unit presents all the current techniques that can be applied for the recycling of the polymers found in WEEE. Both thermal and catalytic recycling methods are presented in detail, along with their advantages and disadvantages. Finally, but importantly, extra attention is paid to pyrolysis as a recycling method for plastics retrieved from WEEE.

### 3.1. Mechanical Recycling

Mechanical or physical recycling refers to the separation and recovery of different types of polymers from the WEEE plastic mixture. Mechanical recycling is the most widely used method for recycling WEEE plastics and requires the preliminary stages of shredding and dismantling after collection followed by washing and granulation to remove impurities from polymers and reduce their size. The manual dismantling of WEEE is a typical “recycling process” in many countries [29]. Adequate sorting is then needed. One of the most widely used processes for sorting polymers is flotation, which is based on separation depending on the density of the material. Pure water is one of the most common flotation media used to separate mainly polyolefins, having a density less than 1 kg/L, from other polymers. Furthermore, salty water can be used to separate styrenic polymers with density within the range 1.0 to 1.1 kg/L from other “heavier” polymers, such as PC, PVC, etc. This heavy fraction is disposed of in landfills or sent to combustors for energy recovery [41,42]. The recovered polymers are sent to re-manufacturing via extrusion or injection molding to obtain virgin polymers with adequate thermo-mechanical properties. While mechanical recycling offers several advantages, including simplicity and cost-effectiveness, it is limited by factors such as polymer degradation, contamination, and material compatibility.

### 3.2. Thermo-Chemical Recycling

**Figure 5:**
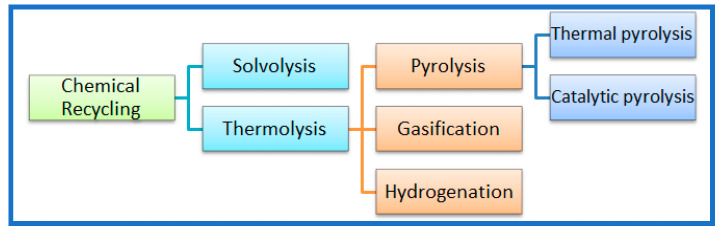
Chemical recycling methods of plastic waste from WEEE [26].

Chemical recycling encompasses various techniques (Figure 5) for depolymerizing WEEE plastics into monomers or low-molecular-weight compounds, which can be used as raw materials for the production of new polymers or chemicals. Specifically, it comprises solvolysis and thermolysis. The former involves the dissolution of the polymers in a solvent, whereas the latter involves heating in an inert atmosphere (e.g., N_2_ atmosphere) in the absence of air or oxygen and includes pyrolysis, gasification, and hydrogenation [26,43]. Pyrolysis, hydrolysis, and solvent-based processes are some common chemical recycling methods employed for WEEE plastics [9,14,23]. Chemical recycling offers the advantage of converting mixed or contaminated plastics into valuable products, overcoming limitations associated with mechanical recycling [44]. However, challenges such as energy consumption, scalability, and product quality need to be addressed for the widespread adoption of chemical recycling technologies. It should be pointed here that in the literature [9], when the chemical recycling of plastics from WEEE is reported, it is usually identified as feedstock recycling, including only thermal and catalytic pyrolysis together with hydrothermal treatment.

#### 3.2.1. Thermal Pyrolysis

Thermal pyrolysis, a thermochemical conversion process, offers a promising solution for the valorization of WEEE plastics by converting them into valuable products such as oils, gases, and char [45,46]. It involves the decomposition of plastics in the absence of oxygen at elevated temperatures. The process typically occurs in a reactor under controlled conditions, with heating rates, residence times, and temperatures influencing the pyrolysis kinetics and product distribution. During thermal pyrolysis, macromolecules in plastics undergo thermal degradation, resulting in the formation of smaller molecules (either liquid or gases) and solid residues [47,48]. Several papers have been published on the pyrolysis of plastics from WEEE (see, for example, [14,18,19,40]), together with patent publications [49,50,51,52,53,54].

Optimizing process parameters is essential to maximize the yield and quality of pyrolysis products. Factors such as temperature, heating rate, residence time, and feedstock composition significantly affect pyrolysis performance [23,26,55]. Higher temperatures generally lead to increased gas and liquid yields but may also promote undesired side reactions such as coke formation. Therefore, a balance must be struck to achieve optimal product yields and quality.

The characterization of pyrolysis products is crucial for assessing their composition, properties, and potential applications. Analytical techniques such as gas chromatography–mass spectrometry (GC-MS), Fourier-transform infrared spectroscopy (FTIR), and elemental analysis are commonly employed to analyze liquid, gas, and solid products [23,56]. The identification of valuable compounds, such as hydrocarbons and aromatics, provides insights into potential applications in fuel production, chemical synthesis, and material recovery.

Fraunhofer UMSICHT developed an innovative thermochemical conversion process for the treatment of WEEE [54]. This process is based on an auger reactor equipped with a heat exchanger. The so-called iCycle^®^ process (Intelligent Composite Recycling) enables the conversion of WEEE fractions at constant and controlled process conditions (the heating up and retention time of feedstock and the stability of process temperature). They found that that a combination of pyrolysis and the subsequent fractional distillation are suitable methods for the isolation of high-purity BTEX fractions and concentrated monocyclic aromatic fractions. The recovery of aromatics from a WEEE nonmetal fraction instead of energetic utilization, however, enables a significant improvement in both economic and ecologic aspects of WEEE recycling.

Thermal pyrolysis offers opportunities for resource recovery from WEEE plastics while mitigating environmental impacts associated with conventional disposal methods. By converting plastics into valuable products, thermal pyrolysis contributes to resource conservation, energy recovery, and waste reduction [46]. Moreover, the production of pyrolysis oils and gases can serve as alternatives to fossil fuels, promoting sustainability and reducing greenhouse gas emissions. At present, the most feasible alternative for pyrolysis oils is their mixture with petroleum streams and further processing in oil refineries; however, refineries are not prone to accept unconventional oils, even less so if such oils are under suspicion for containing halogens and metals. The possibilities of valorizing by pyrolysis the plastic-rich fractions rejected from phone recycling plants have been assessed [18]. Phone waste pyrolysis liquids are a complex mixture of organic compounds (C4–C16), mainly aromatic, with high HHV (34–38 MJ/kg) and contain valuable chemicals such as toluene, styrene, methylstyrene, etc. Almost no Cl and Br of the initial samples are transferred to the liquids (they contain <0.1 wt.%).

Nevertheless, the main difficulty during the direct thermal pyrolysis of plastics retrieved from WEEE is the formation of halogenated (brominated) compounds in the pyrolysis products due to the frequent presence of halogenated flame retardants and mainly BFRs in WEEE. The production of the aforementioned compounds hinders the reuse of the pyrolysis fractions. Therefore, either a “pretreatment” step before pyrolysis (such as various solvent extraction techniques) or during pyrolysis such as co-pyrolysis or even catalytic pyrolysis is often necessary in order to obtain bromine-free products.

For instance, a very common pretreatment method prior to pyrolysis is that of solvent extraction [48], which is traditionally performed via Soxhlet extraction. The latter is considered as an attractive choice because of its simplicity and low-cost despite the fact that it requires a long extractive time and volume of extractive solvents [57,58]. Apart from Soxhlet extraction, another advanced solvent extraction methods that take place requiring a shorter time and lower volumes of solvents have been investigated such as supercritical fluid extraction (SFE), pressurized liquid extraction (PLE), ultrasonic assisted extraction (UAE), and microwave-assisted extraction (MAE) [58].

As already mentioned, apart from the “common” pyrolysis, co-pyrolysis has also been investigated. Co-pyrolysis involves the presence of chemical additives (two or more materials), aiming to improve the quality and quantity of the pyrolysis oils and avoiding the use of catalysts and solvents. During this process, emphasis is given on the synergistic effect of the various materials, which react together during pyrolysis [59]. An interesting study is that of Ma et al. [60], who examined the co-pyrolysis of HIPS (which was brominated flame-retarded with decabromodiphenyl oxide (DDO), along with antimony trioxide (Sb_2_O_3_) as a synergist) in the presence of PP in an attempt to evaluate its effect on the reduction in bromine found in the pyrolysis products. They found that PP led to bromine reduction in pyrolysis oil when compared with its original value (in the absence of PP). As a result, it was observed that the presence of PP had a positive effect on the pyrolysis products and, in the meantime, resulted in a reduced volume of waste, taking into account that more plastic waste (PP) was used as feedstock [59]. Therefore, co-pyrolysis seems to be a promising alternative method.

Other studies have examined the application of a two-step pyrolysis process, with the aim of optimizing the derived pyrolysis products. An indicative example is the work of Ma et al. [61] who applied both single- and two-step pyrolysis to waste computer casing plastic samples. During the single-step pyrolysis, one heating step was applied, reaching a final temperature within the range of 350–600 °C while holding the samples at that temperature for 90 min. On the other hand, during the two-step pyrolysis, two heating steps were applied; the first step included samples being heated up to 350–380 °C and holding this temperature for 15 min, while during the second step, another increase in temperature took place, reaching 500 °C (the samples were held at that temperature for 90 min). It was observed that during the two-step pyrolysis, most of the brominated compounds were found in the liquid fraction of the first step, indicating the effect of the heating steps on the product’s distribution. Therefore, the application of two-step pyrolysis may lead to high-quality pyrolysis oils with low bromine content, enabling their possible use as fuels or chemicals.

#### 3.2.2. Gasification

As already mentioned, thermochemical and chemical processes are the most important treatments for the conversion of plastic waste from EEE into energy and fuels [62,63]. During gasification, the partial oxidation or indirect combustion of polymers occurs, applying high temperatures (up to 1600 °C) and in the presence of oxygen. This method leads to the formation of two main products, CO and H_2_, named as synthesis gas—syngas; this may be used to run a gas engine or may be converted into hydrocarbon fuels via the Fischer–Tropsch process [43,47].

Specifically, aplastic waste fraction derived from electrical sources can be mixed with biomass and successfully recovered via gasification processes. In particular, a high-energy potential syngas, often with a high concentration of hydrogen, can be produced through the thermochemical conversion of obsolete end-of-life plastic materials, while waste products in the form of chars, ashes and slag are formed [63,64].

In other cases, biomass may undergo thermochemical conversion, which consists of several steps: gasification, pyrolysis, a hydrothermal reaction and hydrolysis to convert it into sugars [65]. System alternatives to fossil fuels have been recently considered to fulfil a high demand for energy and, at the same time, to reduce the use of conventional (fossil) fuels. These systems are able to produce syngas from renewable biomass gasification [66].

On the other hand, biomass can be mixed with more than 20% plastic and rubber waste in order to yield highly energy-efficient syngas, thus enabling the recovery waste that would otherwise be sent to landfills [67,68,69]. Alternatively, waste insulating electrical cables (WIECs), after the recovery of the noble metals, can be treated by gasification for energy recovery purposes [70].

A simple and low-cost process was adopted with the aim of causing a high-value modification of biomass, thus producing hydrogen-rich synthesis gas, phenol-rich bio-oil and nanostructured porous carbon materials with high specific surface area through a one-step in situ catalytic pyrolysis at 500 °C [71].

The effect of fly ash-based catalysts, derived by coal gasification, on the pyrolysis of WEEE plastics was investigated in the past by Benedetti and co-workers [72]. In this study, a higher yield of light oil was obtained using fly ash-derived catalysts.

Nevertheless, because condensable liquids or petrochemicals are usually more desirable products, pyrolysis is often favored over gasification, considering the fact that, during gasification, multiple steps are necessary in order to obtain liquid products [43,47].

#### 3.2.3. Hydrogenation

Hydrogenation involves the conversion of large hydrocarbon molecules into lower-molecular-weight products and takes place in hydrogen atmosphere at high pressure (approximately 100 atm) and at moderate temperatures within the range of 150–400 °C [43]. An interesting study is that of Dufaud and Casset (1998), where catalytic hydrogenation took place. Specifically, a silica-alumina-supported zirconium monohydride catalyst was used for the hydrogenation of PE and PP at mild temperatures and pressures [73]. A detailed presentation of the catalytic recycling can be found in the following section.

### 3.3. Catalytic Pyrolysis

Catalytic pyrolysis involves the thermal degradation of plastics in the presence of a catalyst, typically under controlled temperature and pressure conditions. Catalytic pyrolysis has emerged as a promising technology for the valorization of WEEE plastics, converting them into valuable hydrocarbons and reducing environmental impacts. In catalytic pyrolysis, the presence of a catalyst facilitates the decomposition of plastics at lower temperatures, enhances product selectivity, and minimizes undesirable by-products [74]. Catalytic pyrolysis is usually a two-step process that involves the pyrolysis of BFR plastics and the catalytic upgrading of the pyrolysis products. It can be divided into two options [9]: on-line catalytic upgrading (using pyrolysis vapors as raw material) and off-line catalytic upgrading (using pyrolysis oil as raw material). This report provides an overview of the catalytic pyrolysis of WEEE plastics, focusing on process optimization, catalyst selection, product characterization, and potential applications.

The catalyst plays a crucial role in determining the pyrolysis kinetics, product distribution, and quality of the resulting products. Various types of catalysts, including zeolites, metal oxides, and supported metals, have been investigated for the catalytic pyrolysis of WEEE plastics. The choice of catalyst greatly affects the efficiency and selectivity of the pyrolysis process [9]. Zeolites, due to their high surface area and acidity, have shown excellent catalytic activity for the degradation of WEEE plastics [14]. Metal oxides such as MgO, ZnO, and Al_2_O_3_ have also demonstrated catalytic properties, and therefore, they influence the products’ distribution and improve hydrocarbon yields. Supported metal catalysts, including Ni, Pt, and Pd, have been investigated for their ability to enhance cracking reactions and suppress coke formation. Garcia et al. [75] used a fixed bed reactor to investigate the catalytic pyrolysis of high-density polyethylene with hybrid zeolite–MCM-41 mesoporous catalysts and reported a high product yield of light hydrocarbons, mainly C2–C5 alkenes. Aguado et al. [76] used a two-stage pyrolysis–catalysis reaction system to process low-density polyethylene with a zeolite HZSM-5 catalyst. The catalytic pyrolysis of plastic waste collected from waste electrical and electronic equipment in a two-stage reactor system using the Y zeolite and ZSM-5 zeolite has been investigated by Muhammad et al. [77]. They reported that the addition of a zeolite catalyst to the process was mainly dependent on the Si:Al characteristics of the zeolite catalyst used. Zeolite catalyst with a lower Si:Al ratio (Y zeolite) produced a higher conversion of the styrene to other aromatic products, particularly benzene and toluene.

The optimization of process parameters is essential to maximize the yield and quality of the pyrolysis products. Parameters such as temperature, residence time, catalyst loading, and feedstock composition significantly influence pyrolysis performance. Higher temperatures generally result in increased gas and liquid yields but may also promote coke formation and catalyst deactivation. Therefore, an optimal balance of temperature and other parameters is essential to achieve the desired product yields and quality.

The characterization of pyrolysis products is essential to assess their composition, properties, and potential applications. Gas chromatography–mass spectrometry (GC-MS), Fourier-transform infrared spectroscopy (FTIR), and elemental analysis techniques are commonly used to analyze liquid, gas, and solid products. The identification of valuable compounds, such as olefins, aromatics, and hydrocarbons, provides insights into the potential applications of pyrolysis oils as fuels, chemicals, or feedstocks for further processing.

Catalytic pyrolysis offers a sustainable approach for the recovery of resources from WEEE plastics, reducing environmental pollution and conserving valuable materials [78]. By converting plastics into useful hydrocarbons, catalytic pyrolysis mitigates the need for fossil fuel extraction and contributes to the circular economy. Moreover, the valorization of WEEE plastics through pyrolysis helps to address the challenges associated with plastic waste management and promote resource efficiency. Also, as mentioned previously, the presence of catalysts involves a dehalogenation (debromination) effect. This is of paramount importance for the recycling of plastics from WEEE since they usually comprise flame retardants that need to be removed before/during the recycling process. A lot of studies [79,80,81,82,83,84] have investigated various catalysts for their dehalogenation (debromination) efficiency, and two indicative and very recent works are presented in detail.

A recent study of Lopez et al. (2024) [25] studied the catalytic pyrolysis of WEEE plastics, investigating various catalysts, such as bulk metal oxides (Fe_2_O_3_ and CaO), zeolites (ZSM-5 and USY) and hybrid solids by supporting the aforementioned metal oxides on the zeolites. They came to the conclusion that catalytic pyrolysis resulted in reduced halogen content in the pyrolysis oil. The best dehalogenation results were received in the case of the metal-modified zeolites and especially Fe/ZSM-5, which was the most efficient catalyst, as regards the dehalogenation activity and also led to a liquid fraction enriched in monoaromatic hydrocarbons (such as styrene). The aforementioned observations may be attributed to a synergistic effect due to the high Fe dispersion over the zeolite.

In another work [85], the catalytic performance and the debromination efficiency of the Fe-Ni bimetallic MCM-41 catalyst was studied, applying two-stage pyrolysis on waste computer casing plastic samples. They observed that, in the presence of these catalysts, excellent debromination efficiency was achieved. The probable suggested debromination mechanism of the catalysts included different mechanisms: the catalytic cracking of the organobromines, the reaction of loaded metal oxides with HBr/SbBr_3_, and the deposition of organobromines on the surface of the catalysts.

### 3.4. Emerging Technologies

Emerging technologies, including enzymatic degradation, supercritical fluid extraction, and microwave-assisted recycling, hold promise for overcoming the limitations of traditional recycling methods and enabling the efficient recovery of WEEE plastics. These innovative approaches offer opportunities for selective depolymerization, reduced energy consumption, and improved product quality. However, further research and developments are required to optimize these technologies for industrial-scale applications and commercial viability.

Upcycling plastic waste into hydrogen gas is gaining more and more interest, taking into account the importance of hydrogen energy in achieving the carbon neutrality goal [86,87]. Specifically, during the upcycling of plastic waste into hydrogen gas, the breakdown of long hydrocarbon chains takes place, applying various physical or chemical conditions, such as a high temperature and electric field [88]. Generally, the aforementioned thermochemical methods (pyrolysis, gasification, etc.) are the most widely applied methods for plastic-to-hydrogen upcycling. It should be highlighted here that the efficiency of the hydrogen production strongly depends on the processes and conditions applied, including, temperature, time, pressure, atmosphere, the plastic waste used as feedstock, etc. [88,89].

### 3.5. Combined Approaches

Integrated approaches combining mechanical and chemical recycling methods have the potential to enhance the efficiency and sustainability of WEEE plastic recycling. By leveraging the strengths of both mechanical and chemical processes, integrated recycling schemes can maximize material recovery, minimize waste generation, and reduce environmental impacts. Synergistic combinations of technologies, such as cascade processing and hybrid systems, offer opportunities for the comprehensive valorization of WEEE plastics.

A different, combined approach was explored in recent research [90] by applying the microwave co-pyrolysis of biomass and electronic plastic waste, aiming to obtain hydrocarbon fuel. During this work, various process parameters, including the operating temperature, mixture ratio, and the catalyst loading were modeled and optimized. The findings showed that this approach (the microwave co-pyrolysis of biomass with plastic waste) has great potential for energy production and the sustainable conversion of e-waste plastic and biomass residue into fuel while helping to achieve the sustainability goal of reducing global warming potential emissions.

## 4. Discussion

### 4.1. Challenges

Over the last few decades, the WEEE fraction generated among the different continents has increased, and undoubtedly, plastic waste originating from EEE represents a significant waste stream globally. Therefore, the investigation of appropriate recycling methods for plastics’ handling is important. This review provides the reader with all the necessary updated information concerning plastics from WEEE.

Firstly, a definition of WEEE, along with its categories and composition, is presented. Emphasis is given on the hazardous additives that are present in WEEE, including heavy metals, flame retardants, and others. It is highlighted that although the legislation (the RoHS Directive) about the hazardous substances found in WEEE is getting stricter over the last few years, hazardous compounds are found in plastics from EEE, necessitating their careful treatment. Therefore, this study presents various thermal and catalytic recycling methods applied to plastics from WEEE.

Mechanical recycling is often used for the handling of plastic waste originating from EEE, while thermo-chemical recycling methods, including thermal pyrolysis, gasification, and hydrogenation are gaining a lot of interest. Details and examples for the aforementioned techniques are presented; however, more attention is paid to pyrolysis due to the advantages associated with this method.

The catalytic recycling of plastics from WEEE, with the aim of obtaining valuable products, while reducing the halogenated compounds, is also analyzed. The presence of catalysts is of paramount importance since they strongly affect the distribution of the products obtained. Finally, but importantly, combined approaches that can be applied for the recycling of plastics from WEEE are mentioned too.

**Figure 6:**
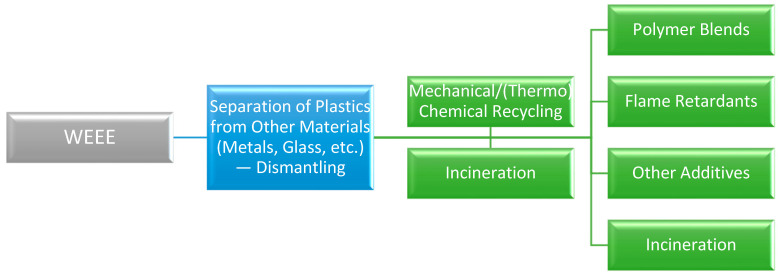
Challenges in the recycling of plastics from WEEE.

Some current challenges in the recycling of the plastic part from WEEE are presented in Figure 6.

The initial debate is about whether mechanical or chemical treatment should be applied. It is suggested these two processes to be used complementarily. In this way, initially, a physical method could be used for sorting different polymers, followed by a thermo-chemical method (i.e., pyrolysis or catalytic cracking) for streams. Therefore, all different streams should be valorized and valuable products could be recovered. The final option, only for materials that cannot be recovered in pure form, could be incineration for energy recovery.

The second point of concern is whether polymer mixtures should be recycled as they are or only after the separation of the individual polymers takes place. The suggestion here is that whenever it is possible, the different polymers included in a blend should be separated and recycled individually. The main reasons for this are that different polymers usually exhibit very different physico-chemical properties, and during thermal degradation, they end up in very different chemical compounds (i.e., aromatics from styrenic polymers, such as HIPS, ABS, hydrocarbons from polyolefins, such as PE, PP, phenols from polycarbonates, PC, etc.). Moreover, sometimes the degradation of one polymer may result in products affecting the decomposition mechanism of another polymer, and also, a specific catalyst type found as the optimum solution for one polymer may not have any effect on the degradation of another polymer type.

Finally, a point of concern is whether flame retardants or other hazardous additives present in the polymers should be separated before thermochemical recycling or not. Since most of these additives may contain harmful compounds (i.e., halogen atoms) [33], they should be removed before the occurrence of the main thermo-chemical recycling process in order to avoid contamination of the final products with halogenated compounds.

### 4.2. Future Perspectives

Despite its potential benefits, thermal pyrolysis faces challenges such as feedstock variability, reactor scalability, and product upgrading. Future research should focus on addressing these challenges through advanced reactor designs, the optimization of process parameters, catalyst integration, product refinement techniques, and the development of circular economy strategies. In addition, more dehalogenation techniques prior to recycling (pyrolysis) should be investigated since the aforementioned solvent-based techniques imply environmental issues that should be taken into account, considering that most of the solvents used are hazardous for the environment. Therefore, over the last few years, research has been focused on searching and applying environmentally friendly green solvents, for instance, alcohols, such as isopropanol or butanol, which show great potential when used for specific types of plastics; yet, a lot of work is needed in order to evaluate their efficiency for various polymer types. Apart from the environmental issues, cost issues should be considered too since the use of large volumes of solvents can restrict their possible application in larger-scale solvent-based methods, for instance, industries, due to their prohibitive cost in such large volumes.

Furthermore, the application of catalytic pyrolysis on a larger scale (e.g., industrially), with the aim of removing the undesirable halogenated (brominated) compounds formed, needs additional research in order to evaluate, for instance, if the selected catalysts are able to be used many times before the formation of coke that may restrict their afterwards use.

Additionally, techno-economic analysis and life cycle assessment studies are needed to evaluate the economic viability and environmental sustainability of thermal pyrolysis for WEEE plastic management. Collaboration among stakeholders, including policymakers, industry players, and research institutions, is essential for accelerating the transition towards sustainable WEEE plastic management. The challenges of the recycling industry in terms of economic and business prospects can be helped by public awareness, private and public partnerships, and the implementation of a sustainable business model.

## 5. Conclusions

Due to the increasing volume of plastics originating in WEEE, in the last few decades, the investigation of appropriate techniques for their recycling has become important. In this review, all current thermal and catalytic recycling methods, with an emphasis on pyrolysis, are presented in detail, along with the challenges that need to be handled. The thermal pyrolysis of plastics from waste electric and electronic equipment (WEEE) offers a promising pathway for resource recovery and environmental sustainability. Through process optimization and product characterization, thermal pyrolysis demonstrates its potential to convert WEEE plastics into valuable products, thereby reducing waste generation and promoting circular economy principles.

Nevertheless, the direct thermal pyrolysis of plastics from WEEE often leads to the formation of undesirable halogenated and mainly brominated compounds because of the presence of flame retardants in the plastic fraction of WEEE. For this reason, pretreatment methods are usually applied either before (e.g., solvent-based methods) or during recycling–pyrolysis, such as co-pyrolysis, which depends on the synergistic effect of some different materials that react together during it, or even catalytic pyrolysis since catalysts can strongly affect the product’s distribution.

Specifically, the catalytic pyrolysis of plastics from waste electric and electronic equipment (WEEE) presents a promising strategy for resource recovery and environmental sustainability. By optimizing process parameters, selecting appropriate catalysts, and characterizing pyrolysis products, significant progress has been made towards the development of efficient pyrolysis technologies. Future research should focus on further improving catalyst performance, enhancing product selectivity, and exploring novel applications for pyrolysis oils and gases.

## Data Availability

No new data were created.
